# Evaluation of aerosol IL-2 in sarcoma patients with lung metastases for future combination therapy with infused natural killer cells

**DOI:** 10.1186/2051-1426-1-S1-P251

**Published:** 2013-11-07

**Authors:** Aung Naing, Pete Anderson, Sergei Guma, Hsuan-Chu Chien, Lacey MM McQuinn, Joshua P Hein, Ralph G Zinner, Eugenie S Kleinerman, Nancy Gordon

**Affiliations:** 1 Investigational Cancer Therapeutics, MD Anderson Cancer Center, Houston, TX, USA; 2 Department of Hematology-Oncology and Bone Marrow Transplant, Levine Children's Hospital, Charlotte, NC, USA; 3 The Children's Cancer Hospital, Division of Pediatrics, MD Anderson Cancer Center, Houston, TX, USA; 4 Department of Cancer Biology, MD Anderson Cancer Center, Houston, TX, USA

## 

To circumvent significant toxicities associated with high dose systemic IL-2 treatment, organ-specific delivery such as inhalational IL-2 has been studied. Our pre-clinical data demonstrated therapeutic efficacy of aerosol IL-2 in a human OS mouse model with no toxicity. The number of OS lung metastases in the aerosol IL-2 treated group was significantly less than the aerosol PBS control group (p = 0.03). IHC of OS lung nodules from mice treated with aerosol IL-2 showed a significant increase in apoptosis measured by TUNEL compared to the aerosol PBS group (p= 0.003). Aerosol IL-2 significantly increased proliferation of local NK cells in the lungs compared with aerosol PBS group (p= 0.03 and p=0.007 at 24 and 72 hours respectively). There was no proliferation of NK cells in other organs and no effect on T reg. Since sarcoma metastasizes to the lung, aerosol delivery is a reasonable approach. The study is to determine the safety of aerosol IL-2 in patients > 12 yrs. (with emphasis on those < 18years) with lung metastases to establish the maximum tolerated dose (MTD) to use for combination therapy with infused natural killer (NK) cells in patients with Osteosarcoma (OS) lung metastases. Patients with lung metastases only received aerosolized IL-2 for 3 consecutive weeks (21 day cycle) for 2 cycles with 1-week rest between cycles. Each patient is educated to administer the treatment for the first time in the hospital. For further home treatments, remote spirometries as well as pulse oximetry are recorded before each treatment. Data is downloaded on a web portal for investigators to access and captured into the patient's medical record. Results: Four patients (age 18-66); all male; 3 with primary diagnosis of Ewing's Sarcoma and 1 with Osteosarcoma. Side effects were grade 1 fatigue (n= 1), grade 1 cough (n= 2), and grade 1 wheezing (n=1.). None of these adverse events have been attributed to the IL-2 therapy since symptoms resolved without intervention while on therapy. Serum levels of IL-2 determined by ELISA immune assay in the first three patients were undetectable but low level in the pt from the highest dose thus far. In conclusion, clinical evaluation of aerosol IL-2 therapy to determine feasibility and safety will potentially benefit patients with OS who succumb almost always due to lung disease and allow future combination therapy with infused NK cells.

